# Pharmacokinetics of Bexagliflozin After Intravenous and Oral Administration in Cats

**DOI:** 10.1111/jvp.70072

**Published:** 2026-04-11

**Authors:** Yogini Patel, Sheeva Bhattarai, Céline E. Toutain

**Affiliations:** ^1^ Elanco Animal Health Yarrandoo R&D Centre Kemps Creek New South Wales Australia; ^2^ Elanco Animal Health Sèvres France

**Keywords:** bexagliflozin, cat, diabetes mellitus, pharmacokinetics, SGLT2 inhibitor

## Abstract

Bexagliflozin, a selective sodium–glucose cotransporter 2 (SGLT2) inhibitor, has been developed for feline diabetes mellitus. This study characterized bexagliflozin pharmacokinetics in healthy cats after single intravenous and oral administrations. Sixteen adult cats received a 1 mg/kg intravenous solution and a 15 mg oral tablet (Bexacat) in a two‐period study under fasted conditions. Serial plasma samples were collected up to 72 h and analyzed by a validated LC–MS/MS method (calibration range 1–2000 ng/mL). Pharmacokinetic parameters were derived by non‐compartmental analysis. Following intravenous dosing, bexagliflozin showed a low plasma clearance of 348 mL/h/kg. The volumes of distribution during the terminal phase (*V*_z_) and at steady‐state (*V*_SS_) were 3.5 and 1.7 L/kg, respectively. The terminal half‐life was 7.0 h. After oral dosing of the tablet, absorption was rapid (median *T*_max_ 1 h; range 0.5–4 h), with *C*_max_ of 1780 ng/mL and a terminal half‐life of 6.9 h. Absolute bioavailability was 78% (90% confidence interval: 73%–83%). Exposure was comparable between males and females after both routes (AUC ratios ~97%–103%). Treatments were well tolerated. These pharmacokinetic data indicate bexagliflozin exhibits rapid absorption, high oral bioavailability, and a half‐life compatible with once‐daily dosing in cats.

## 1 Introduction

Sodium–glucose cotransporter 2 (SGLT2) inhibitors represent a significant advancement in the management of type 2 diabetes mellitus in humans (Scheen 2015; Rieg and Vallon 2018). These drugs exert their therapeutic effect by selectively inhibiting SGLT2, the primary protein responsible for glucose reabsorption in the renal proximal tubules (Vallon et al. 2011; Abdul‐Ghani et al. 2011). This inhibition promotes increased urinary glucose excretion, thereby lowering blood glucose levels independently of insulin action (Sano et al. 2020). Such a mechanism offers distinct advantages over traditional insulin secretagogues or insulin sensitizers, notably by mitigating the risk of hypoglycemia (Vallon and Thomson 2017; Horii et al. 2020). Bexagliflozin, a selective SGLT2 inhibitor, has demonstrated significant blood glucose‐lowering efficacy and a well‐tolerated safety profile in human clinical trials (Kamrul‐Hasan et al. 2025; Allegretti et al. 2019). Its favorable characteristics, including minimal off‐target effects, have prompted its investigation in veterinary species, particularly for the management of diabetes mellitus in cats (Scott‐Moncrieff 2024). Feline diabetes mellitus shares key pathophysiological similarities with human type 2 diabetes mellitus, such as insulin resistance and β‐cell dysfunction, making cats a relevant species for evaluating SGLT2 inhibitors (Cook and Behrend 2025). Current management of feline diabetes predominantly relies on daily insulin injections, which can present challenges for owners (e.g., administration burden, risk of hypoglycemia) and may result in suboptimal glycemic control in some cats. The development of an oral agent like bexagliflozin offers the potential to significantly improve the therapeutic landscape for this common feline endocrine disorder (Benedict et al. 2022; Hadd et al. 2023). Metabolism and disposition of bexagliflozin have been investigated in rats, monkeys, and humans (Zhang et al. 2020). The objective of this study was to characterize the pharmacokinetic profile of bexagliflozin in healthy adult cats following single intravenous and oral administration.

## 2 Materials and Methods

### 2.1 Guidelines, Animal Welfare, and Regulatory Standards

The study presented in this manuscript was performed according to the Principles of Good Laboratory Practice (OECD 1998) and the Guideline on conduct of pharmacokinetic studies in target animal species (EMA 2024). This was a laboratory study performed at Elanco's test facility located in Australia, in compliance with the Australian code for the care and use of animals (National Health and Medical Research Council 2013). It was conducted under the Australian Pesticides and Veterinary Medicines Authority Small‐scale Trials Permit PER7250 and the protocol was reviewed and approved by the Elanco Animal Ethics Committee. The bioanalytical method was validated according to the US and EU bioanalytical method validation guidelines (US FDA 2018; EMA 2012).

### 2.2 Study Design

Sixteen domestic cats from Elanco's cat colony (eight males and eight females) aged 12 months to 8.7 years and weighing 3.1–5.7 kg were selected for inclusion in the study after evaluation for suitability by a veterinary examination that included clinical pathology and body weight. Cats were acclimatized to study conditions for 7 days. They were housed indoors, and temperature and humidity were monitored in the facility. They were fed once daily with an appropriate ration of a commercial feline feed (primarily Advance Adult chicken with rice) and water was available ad libitum. On the day of treatment administration and for the duration of blood collection, cats were housed individually, in order to avoid cross‐contamination between animals and to monitor for potential adverse events. Cats were observed for general health, behavior, and appetite at least once daily throughout the study. A final veterinary examination was conducted at the end of the in‐life phase before cats were returned to the colony upon completion of the study.

Each cat received two treatments with a washout period of 2 weeks. During the first week of the study, all 16 cats were treated orally with a single 15 mg bexagliflozin tablet (commercial formulation, Bexacat; Elanco Animal Health). Approximately 3 mL of water was given after tablet administration and the mouth checked to ensure the tablets had been swallowed. In the third week of the study, all cats were treated intravenously at a dose of 1 mg/kg with a 1.5 mg bexagliflozin/mL solution (70% water, 30% PEG400) as a bolus in the cephalic vein via a catheter. The catheter was flushed with approximately 1 mL of saline after injection. The exact administered intravenous dose was calculated by weighing individual syringes before and after administration to determine the amount administered. This value was divided by the intravenous formulation's density to obtain the administered volume. Finally, the volume was multiplied by the drug concentration and divided by the animal's body weight to derive the exact administered dose.

For both the oral and intravenous treatments, cats were fasted overnight (approximately 13 h) prior to dosing, and they were fed their daily ration of food approximately 2 h after treatment administration, following the 2‐h blood collection. Blood samples (1 mL) were collected into EDTA tubes prior to treatment and after treatment at 15 and 30 min and at 1, 2, 4, 8, 12, 24, 48, and 72 h. Samples were centrifuged at 3000 × *g* for 15 min at 4°C and collected plasma samples were stored frozen at approximately −20°C until analysis.

### 2.3 Analysis of Bexagliflozin in Plasma

Plasma samples were analyzed to determine the concentration of bexagliflozin using a validated method. For sample preparation, a 100 μL aliquot of standards, quality control samples, or study plasma samples was transferred into microcentrifuge tubes. To the double blank sample, 40 μL of methanol was added. For all other samples, 40 μL of the internal standard (0.15 μg/mL ^13^C‐bexagliflozin in methanol) was added. Each sample then received 300 μL of acetonitrile, followed by vortex mixing and centrifugation (17,000 × *g*, 15 min, 4°C). A 200 μL aliquot of the supernatant was transferred to a 96‐well collection plate and dried under a stream of nitrogen at 40°C. After drying, 300 μL of methanol was added to the collection plate, vortex‐mixed, and then a 10 μL aliquot was injected into the LC–MS/MS system.

Chromatographic separation was achieved using a Phenomenex Kinetex C18 (50 × 2.1 mm, 1.7 μm) high‐performance liquid chromatography column maintained at 40°C. Elution was performed at a flow rate of 750 μL/min using a gradient method. Mobile phase A consisted of 0.1% formic acid in water, and mobile phase B was 0.1% formic acid in a 50:50 (v/v) acetonitrile:methanol mixture. The gradient began at 30% B, increased to 60% B over 1.70 min, then to 95% B at 1.75 min, and was held at 95% B until 2.50 min. At 2.55 min, the gradient returned to 30% B, with a total analysis run time of 4 min.

Detection was performed using a Sciex 6500 Q‐Trap mass spectrometer equipped with a Turbo Ion Spray source operating in positive ion mode. Multiple reaction monitoring transitions were monitored at m/z 482 → 370 for bexagliflozin and m/z 488 → 169 for the ^13^C‐bexagliflozin internal standard. Both bexagliflozin and the internal standard exhibited retention times of approximately 2.0 min. Quantification was achieved using quadratic regression with 1/*x* ^2^ weighting, covering a concentration range from 1 to 2000 ng/mL. Each analytical run included duplicate standard curves and at least six quality control samples at concentrations of 1.00, 3.00, 80.0, 1600, and 5000 ng/mL (diluted 1:20). Chromatograms were integrated using Analyst Software (v1.7.1) and data were processed in Watson laboratory information management system (v7.6).

### 2.4 Pharmacokinetic Analysis

Pharmacokinetic parameters were calculated for individual animals using non‐compartmental analysis with the software Phoenix WinNonlin (version 8.6; Certara L.P., Princeton, NJ, USA). The peak plasma concentration (*C*_max_) and time to peak concentration (*T*_max_) for the oral administration group were determined as observed values. The terminal half‐life (*T*_1/2_) was calculated by log‐linear regression over a suitable time interval, employing the best fit method as implemented in Phoenix WinNonlin.

The area under the plasma concentration–time curve (AUC) was determined using the linear trapezoidal linear interpolation method. Specifically, AUClast represented the area from time zero to the last quantifiable plasma concentration, and AUC0–24 was calculated from time zero to 24 h. The area under the concentration–time curve from time zero to infinity (AUCinf) was derived as the sum of AUClast and the extrapolated area from the last observed time point to infinity, calculated by log‐linear extrapolation using the terminal slope. The mean residence time (MRT) was calculated as the ratio of the area under the first moment curve (AUMC) to AUCinf.

Following intravenous administration, the plasma concentration at time zero (*C*_0_) was determined for each animal by back‐extrapolation from a linear regression of the first two time points. Total plasma clearance (CL), defined as Dose/AUCinf, the volume of distribution during the terminal phase (*V*_z_), calculated as CL × *T*_1/2_/ln2, and the volume of distribution at steady‐state (*V*_SS_), calculated as CL × MRT, were also determined.

For bioavailability and sex effect evaluation, AUC ratios were calculated using the bioequivalence module of Phoenix WinNonlin on natural log‐transformed dose‐normalized AUC obtained from the non‐compartmental analysis. A linear mixed effects model was employed, with route of administration (for bioavailability) or sex (for sex effects) as fixed terms and the cat as a random term. The average type of bioequivalence setting was selected. For the absolute bioavailability calculation, a crossover type of study was selected (comparing the same animals after oral and intravenous administration) with the intravenous formulation set as the reference. For the sex effect evaluation, a parallel type of study was selected (comparing different animals) with males set as the reference group. The mean ratios and their corresponding 90% confidence intervals (CIs) were calculated.

## 3 Results

### 3.1 Method Validation

The analytical method for the determination of bexagliflozin in cat plasma by LC–MS/MS was successfully validated. The assay demonstrated linearity over a concentration range of 1–2000 ng/mL, with the calibration function employing quadratic regression with 1/*x* ^2^ weighting, yielding a coefficient of determination (*R* ^2^) > 0.99.

Accuracy and precision were rigorously assessed. For intra‐batch evaluations, accuracy, expressed as percentage bias, ranged from −12.0% to 6.0%, and precision (coefficient of variation, CV%) did not exceed 9.4% across all quality control levels (1.00, 3.00, 80.0, and 1600 ng/mL). Inter‐batch assessments showed accuracy ranging from −6.3% to 3.0%, with CV% no greater than 8.2%. All results were within the accepted criteria of ±15% (±20% at the lower limit of quantification [LLOQ]) for both accuracy and precision.

Furthermore, dilution integrity was confirmed, as samples with concentrations exceeding the calibration range could be diluted 20‐fold with blank control cat plasma and quantified with acceptable accuracy and precision. The mean accuracy (% bias) and precision following a 20‐fold dilution of quality control samples fortified at 5000 ng/mL were −1.2% and 3.6%, respectively.

The mean extraction recovery of bexagliflozin at 3.00, 80.0, and 1600 ng/mL concentrations was consistently in the range of 106%–119%. Similarly, the overall mean recovery of the internal standard was in the range of 114%–117%. Evaluation of matrix effects revealed no significant interference across six independent sources of matrix. The CV% of the internal standard‐normalized matrix factor did not exceed 6.7% for the low concentration level and 1.9% at the high concentration level, meeting the acceptance criterion of ≤ 15%.

Stability of bexagliflozin was confirmed under various conditions simulating sample handling and storage. Bexagliflozin was stable in cat plasma for at least three freeze–thaw cycles (storage at approximately −20°C), for at least 24 h at ambient room temperature (benchtop stability), and for at least 4 months during long‐term storage at −20°C. Stability was also demonstrated in K_2_EDTA whole blood for at least 2 h at both ambient temperature and on wet ice prior to centrifugation. Solution stability studies confirmed that bexagliflozin stock solution (1 mg/mL in methanol) was stable for at least 78 days at 4°C and 24 h at room temperature, while its working solution (70:30 v/v acetonitrile/water) remained stable for at least 78 days at 4°C. The internal standard stock solution (1 mg/mL in methanol) was stable for at least 82 days at 4°C, and its working solution (0.15 μg/mL in methanol) for at least 144 days at 4°C.

The method exhibited high specificity, with no significant interference from endogenous components observed at the retention times of bexagliflozin or the internal standard. Assay carryover was assessed by injecting blank plasma samples immediately following the highest calibration standard (2000 ng/mL). The response in these blanks was 0% relative to the LLOQ for bexagliflozin and 0% for the internal standard, within the acceptance limits of < 20% and 5%, respectively, confirming that carryover was negligible.

### 3.2 Pharmacokinetics

Plasma concentrations of bexagliflozin were successfully determined in all collected specimens with this validated method. Pre‐dose samples for both oral and intravenous administration consistently showed concentrations below the lower limit of quantification (< LLOQ).

**FIGURE 1:**
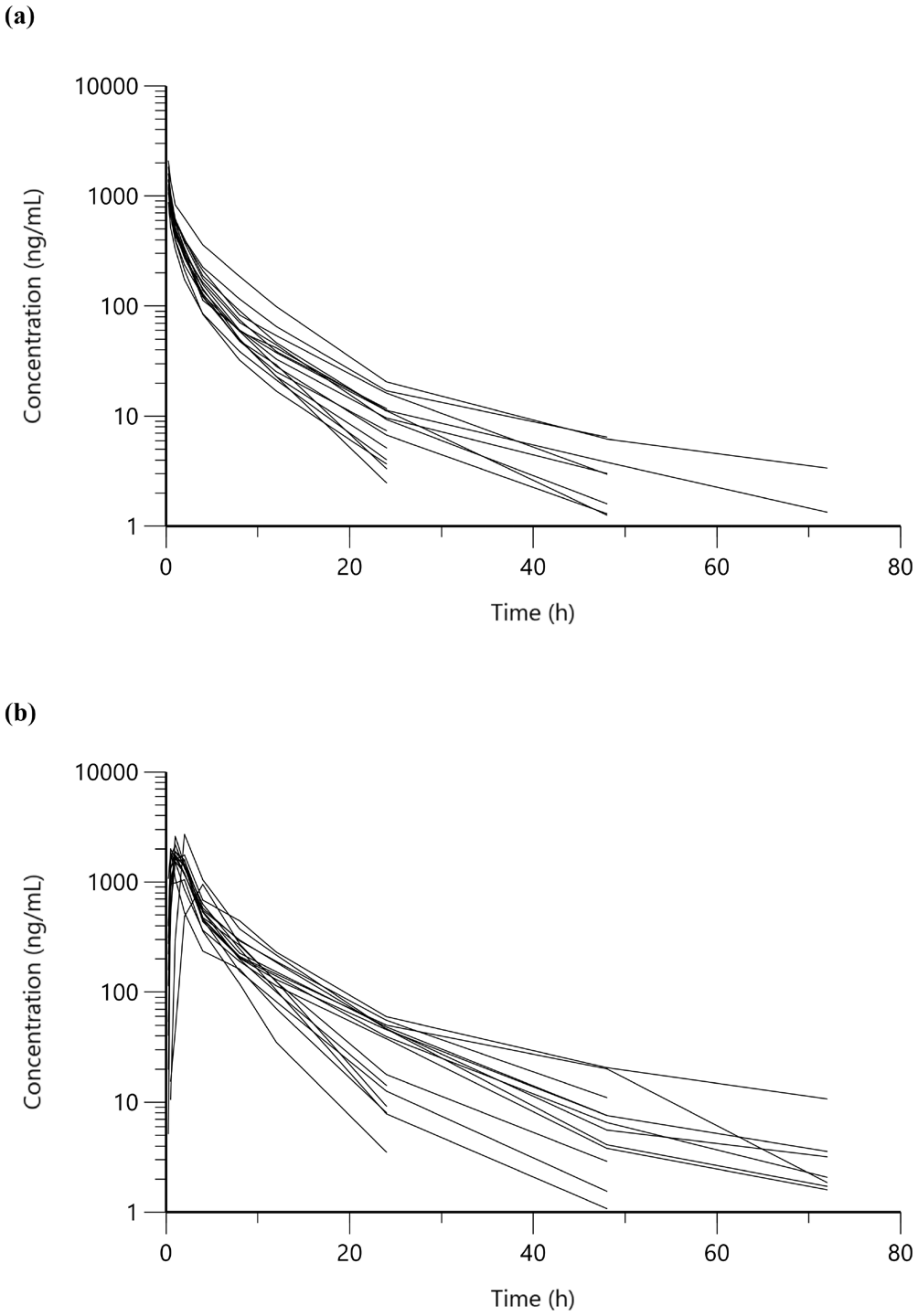
Individual plasma concentration–time profiles of bexagliflozin in cats following a single intravenous administration of 1 mg/kg solution or a single oral administration of a 15 mg tablet. Each line represents an individual cat. (a) Intravenous and (b) oral.

The plasma concentration–time profiles of bexagliflozin following intravenous (1 mg/kg) and oral administration (15 mg) in fasted cats are illustrated in Figure 1, with the corresponding pharmacokinetic parameters summarized in Table 1. All cats were exposed to the treatment, and quantifiable plasma concentrations were observed until at least 24 h post‐dose for both routes of administration, with some quantifiable but low concentrations also noted at 48 and 72 h post‐treatment.

**TABLE 1:** Summary of pharmacokinetic parameters of bexagliflozin in cats following a single intravenous administration of 1 mg/kg solution or a single oral administration of a 15 mg tablet.

	Intravenous (1 mg/kg)	Oral tablet (15 mg)
Exact dose (mg/kg)	1.02 ± 0.02 (0.99–1.07)	3.58 ± 0.75 (2.63–4.84)
*T*_max_ (h)	—	1 (0.5–4)
*C*_0_ (ng/mL)	2070 (26.4)	—
*C*_max_ (ng/mL)	—	1780 (27.4)
*C*_max_/D (ng/mL)	—	507 (29.0)
AUC0–24 (h*ng/mL)	2810 (30.2)	7320 (26.0)
AUC0–24/D (h*ng/mL)	2750 (29.6)	2090 (29.0)
AUCinf (h*ng/mL)	2930 (32.4)	7800 (29.2)
AUCinf/D (h*ng/mL)	2870 (31.9)	2230 (31.3)
CL or CL/F (mL/h/kg)	348 (31.9)	449 (31.3)
*V*_SS_ (mL/kg)	1660 (20.1)	—
*V*_z_ or *V*_z_/F (mL/kg)	3530 (32.7)	4450 (38.2)
MRT (h)	4.76 (30.6)	6.32 (33.1)
*T*_1/2_ (h)	7.03 (46.8)	6.87 (53.1)

*Note:* Data are presented as geometric means (geometric CV%) except for the exact dose, which is given as arithmetic mean ± SD (range) and *T*_max_ as median (range).

All pharmacokinetic parameters presented are based on geometric means, deemed most appropriate given that these parameters are assumed to follow a log‐normal distribution. *T*_max_, being a discrete variable, is reported as a median. After the oral administration, the interindividual variability for *C*_max_ and AUCs, measured by CV%, was moderate (< 30% in general).

Following intravenous administration, actual doses ranged from 0.99 to 1.07 mg/kg, with a mean ± standard deviation (SD) of 1.02 ± 0.02 mg/kg. Bexagliflozin concentrations declined bi‐exponentially after intravenous administration, with a rapid initial distribution phase followed by a longer elimination phase. The terminal half‐life (*T*_1/2_) was 7.03 h, and the mean residence time (MRT) was 4.76 h. Total plasma clearance (CL) was 348 mL/h/kg (5.8 mL/min/kg), while the volume of distribution during the terminal phase (*V*_z_) and the volume of distribution at steady‐state (*V*_SS_) were 3.5 and 1.7 L/kg, respectively. The percentage of extrapolated AUC (%AUCextra) was < 1.2%, indicating that the sampling schedule effectively characterized the concentration–time profiles.

Following oral administration, actual doses ranged from 2.63 to 4.84 mg/kg, with a mean ± SD of 3.58 ± 0.75 mg/kg. Maximum plasma concentrations were achieved within 0.5–4 h, with a median *T*_max_ of 1 h. The mean *C*_max_ was 1780 ng/mL, and the mean dose‐normalized *C*_max_ (to 1 mg/kg) was 507 ng/mL. The oral *T*_1/2_ was 6.87 h, and the MRT was 6.32 h. The percentage of extrapolated AUC (%AUCextra) was < 0.5% after oral administration.

**FIGURE 2:**
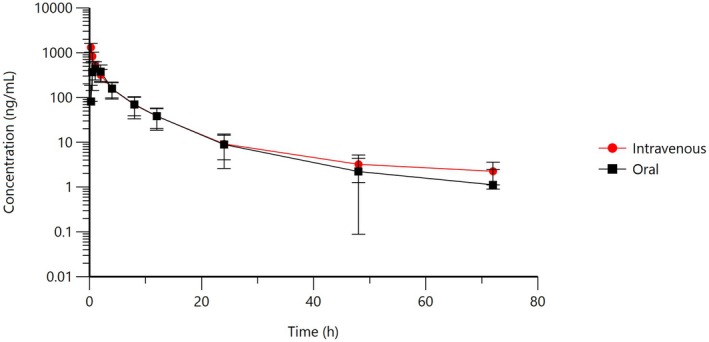
Mean (±standard deviation) dose‐normalized (to 1 mg/kg) plasma concentration–time profiles of bexagliflozin in cats following a single intravenous administration of 1 mg/kg solution or a single oral administration of a 15 mg tablet.

Mean plasma dose‐normalized concentrations, sorted by route of administration, are presented in Figure 2. The absolute bioavailability of bexagliflozin from the tablet formulation, compared to the intravenous solution, was 78% (Table 2) based on AUC parameters.

**TABLE 2:** Absolute bioavailability of bexagliflozin in cats following oral administration relative to intravenous administration, based on dose‐normalized AUC.

Parameter	Reference	Test	Ratio (%Ref.)	90% CI
Ln(AUClast/D)	Intravenous	Oral	78.1	72.5–84.0
Ln(AUCinf/D)	Intravenous	Oral	77.5	72.0–83.4

**FIGURE 3:**
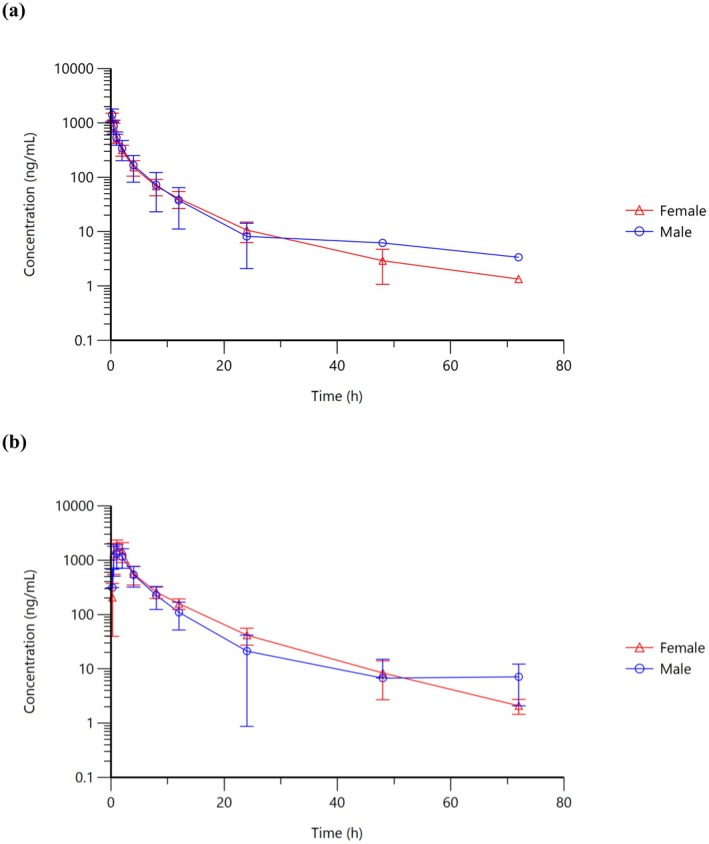
Mean (±standard deviation) plasma concentration–time profiles of bexagliflozin in male and female cats following a single intravenous administration of 1 mg/kg or a single oral administration of a 15 mg tablet. (a) Intravenous and (b) oral.

Mean plasma concentrations, sorted by route of administration and sex, are presented in Figure 3. The sex effect evaluation demonstrated that exposure to bexagliflozin was comparable between male and female cats. After both intravenous and oral administration, the AUC ratios for females to males were approximately 102% and 97%, respectively (Table 3). The confidence intervals for these ratios contained 100%, indicating no significant differences between the sexes.

**TABLE 3:** Evaluation of sex effect on bexagliflozin exposure in cats following intravenous and oral administration, based on dose‐normalized AUC ratios (male vs. female).

Route	Parameter	Reference	Test	Ratio (%Ref.)	90% CI
IV	Ln(AUClast/D)	Male	Female	102.8	77.5–136.3
	Ln(AUCinf/D)	Male	Female	102.12	76.9–135.6
Oral	Ln(AUClast/D)	Male	Female	97.0	73.7–127.7
	Ln(AUCinf/D)	Male	Female	96.8	73.3–127.8

## 4 Discussion

The bioanalytical method to quantify bexagliflozin in cat plasma samples was successfully validated for linearity, selectivity, accuracy, precision, recovery, and carryover according to international regulatory guidelines (US FDA 2018; EMA 2012). The protein precipitation method yielded consistent results across a wide calibration range (1.00–2000 ng/mL), with no significant matrix effects. In addition, stability was demonstrated under all relevant sample handling and storage conditions. The validated method was therefore considered robust, reliable, and fully fit‐for‐purpose for supporting pharmacokinetic studies in cats.

This study presents the first comprehensive assessment of the pharmacokinetics of bexagliflozin in cats. Both intravenous (1 mg/kg) and oral (15 mg) administrations were well tolerated, with no serious adverse events reported during the study period. Although minor adverse events, such as vomiting and diarrhea, were observed, they were deemed unrelated to the treatment, primarily due to their timing, occurring more than 24 h post‐administration. Four events were possibly associated with the treatment: two cats exhibited liquid feces following the oral dose, one cat showed liquid feces on the day of intravenous treatment, and another cat vomited approximately 12 h after receiving the intravenous dose. All adverse events were transient and resolved without any treatment.

Diarrhea is a recognized adverse event for SGLT inhibitors, particularly those with SGLT1 inhibition. This is well‐documented for dual SGLT1/2 inhibitors, where SGLT1 inhibition is strongly implicated in the onset of diarrhea (He et al. 2020; Lehmann and Hornby 2016; Rieg and Vallon 2018). SGLT1 serves as a major transporter in the small intestine for carbohydrate absorption; when inhibited, unabsorbed carbohydrates reach the colon, undergo fermentation, and increase fluid secretion, which is the physiological mechanism underlying diarrhea. Regulatory data and clinical trials consistently report higher rates and severity of diarrhea with dual SGLT1/2 inhibitors compared to SGLT2‐selective agents (Teo et al. 2022; He et al. 2019; Tsimihodimos et al. 2018).

Selectivity of bexagliflozin was evaluated in transfected Cos‐7 cells overexpressing feline SGLT1 or SGLT2, measuring sodium‐dependent uptake of the radiolabeled non‐metabolizable substrate, α‐methyl‐D‐[U‐14C]glucopyranoside, as a function of bexagliflozin concentration in 0% or 10% cat plasma. Bexagliflozin inhibited both transporters dose‐dependently, with greater potency for SGLT2 (IC_50_ 28 pM without plasma; 412 pM with 10% plasma). Its effects on SGLT1 were less pronounced (IC_50_ 3.2 and 23.8 nM under the same conditions), yielding SGLT2/SGLT1 selectivity ratios of 114 (0% plasma) and 57.8 (10% plasma). This selectivity profile provides a basis for understanding the adverse events associated with bexagliflozin.

For a drug administered intravenously, body clearance is defined by the relationship CL_body_ = cardiac output × *E*_body_, where *E*_body_ represents the overall extraction ratio. With a nominal cardiac output of approximately 146 mL/kg/min for a 3 kg cat, the mean clearance of bexagliflozin observed in this study was calculated to be 348 mL/h/kg (equivalent to 5.8 mL/min/kg), resulting in an overall extraction ratio of 4%. This clearance rate is considered low (Toutain and Bousquet‐Mélou 2004a). Furthermore, the relatively large volume of distribution at 3.5 L/kg, combined with the calculated half‐life *T*_1/2_ = ln 2 × Vd/CL = ln 2 × (3.5 L/kg)/(0.348 L/h/kg) = 7 h, corresponds with the observed half‐life of approximately 7 h in the study. The terminal half‐life of bexagliflozin suggests minimal accumulation potential, a conclusion supported by both theoretical and empirical evidence. From a theoretical standpoint, for drugs administered daily, a terminal half‐life shorter than 12 h generally yields an accumulation ratio of < 1.3 (Toutain and Bousquet‐Mélou 2004b). Data from a 26‐week safety study conducted at doses of 1, 3, and 5× the therapeutic dose demonstrated no significant increases in exposure upon repeated dosing, as indicated by AUC ratios on study days 21 and 175 compared to baseline (study day 0), all of which remained below 1 with a terminal half‐life of approximately 5 h (US FDA 2022). These findings confirm the absence of accumulation with long‐term daily dosing of bexagliflozin.

Mean residence time (MRT) provides a model‐independent estimate of the average time drug molecules reside in the body. As expected, MRT after oral dosing includes the absorption process, whereas MRT after intravenous dosing reflects only distribution and elimination. In cats, MRT was 4.76 h after intravenous dosing and 6.32 h after oral dosing. The difference between these MRTs gives the mean absorption time (MAT = MRToral − MRTiv), which was 1.56 h. The relatively short MAT for bexagliflozin in cats indicates a rapid and efficient oral absorption.

After oral administration, the median *T*_max_ of 1 h was consistent with a rapid onset of efficacy. In a field trial involving cats newly diagnosed with diabetes (Hadd et al. 2023), the mean blood glucose concentration decreased rapidly during the 8 h after initiation of treatment. The action of bexagliflozin was apparent after administration of the first dose, as detected by a prompt and reproducible decrease in blood glucose concentration measured over the course of an 8‐h, 5‐sample evaluation.

In the present study, the bioavailability was high in fasted conditions (approx. 80%) but was not investigated in fed conditions. In a preliminary pharmacokinetic study using a near‐final formulation, exposure was slightly lower in fed conditions but glucosuria was similar (Elanco internal data, not shown). The 15 mg fixed dose was selected to simplify administration and enhance owner compliance by providing a convenient dosing regimen of one tablet daily per cat. Under field conditions, the bexagliflozin 15 mg tablet was safe and efficacious across the population's weight range when administered orally either with or without food, reflecting practical usage conditions (Hadd et al. 2023).

## 5 Conclusion

This study characterized the pharmacokinetic profile of bexagliflozin in healthy adult cats following single intravenous and oral administrations. Oral administration resulted in rapid absorption and a high absolute bioavailability of 78% in fasted cats. The terminal half‐life of approximately 7 h and mean residence time values indicate a pharmacokinetic profile compatible with a once‐daily dosing regimen. Furthermore, the absence of drug accumulation in long‐term studies supports its suitability for chronic administration. Bexagliflozin was well tolerated in this study, and no significant sex‐based differences in exposure were observed.

## Author Contributions

**Yogini Patel:** bioanalysis, writing – review and editing. **Sheeva Bhattarai:** study execution, writing – review and editing. **Céline E. Toutain:** project administration, study design, analysis of the results, writing – review and editing.

## Funding

The authors have nothing to report.

## Ethics Statement

The authors confirm that the ethical policies of the journal, as noted on the journal's author guidelines page, have been adhered to and the appropriate ethical review committee approval has been received. The authors confirm that they have adhered to Australian standards for the protection of animals used for scientific purposes.

## Conflicts of Interest

All authors are current employees of Elanco Animal Health.

## Data Availability

Research data are not shared. The data that support the findings of this work are proprietary to Elanco Animal Health.
